# Self-evaluation in schizophrenia: an fMRI study with implications for the understanding of insight

**DOI:** 10.1186/1471-244X-12-106

**Published:** 2012-08-09

**Authors:** Nicholas J Bedford, Simon Surguladze, Vincent Giampietro, Michael J Brammer, Anthony S David

**Affiliations:** 1 Department of Psychosis Studies, Institute of Psychiatry, King’s College London, London, UK; 2 Department of Neuroimaging, Institute of Psychiatry, King’s College London, London, UK

**Keywords:** Insight, Schizophrenia, fMRI, Self-awareness, Medial-frontal cortex

## Abstract

**Background:**

Lack of insight is a core feature of schizophrenia and is associated with structural brain abnormalities. The functional neuroanatomy of insight has only recently been investigated. When people evaluate their personality traits compared to those of another, activation is seen in central midline structures (CMS) of the brain. This study set out to compare cerebral activation in schizophrenia patients versus controls during a self-evaluation task which included positive and negative traits as well as mental and physical illness terms.

**Methods:**

Eleven schizophrenia patients and 8 healthy controls, matched for age were studied. Insight was assessed using the Schedule for the Assessment of Insight-expanded version (SAI-E). FMRI data were obtained with a 1.5 Tesla GE system and interactions between participant group, self versus other, significant at the cluster level, were recorded.

**Results:**

Significant hypoactivation in the medial superior frontal gyrus (dorsomedial prefrontal cortex) was observed in patients vs. controls during self-evaluation of all traits combined. A second cluster of hypoactivation in the posterior cingulate was also detected. When the response to individual traits was explored, underactivation in other frontal regions plus right inferior parietal lobule emerged and this tended to correlate, albeit weakly with lower insight scores. Further, there were areas of hyperactivation relative to controls in anterior cingulate, frontal and parietal regions (especially precuneus) which showed moderate inverse correlations with insight scores.

**Conclusions:**

We have demonstrated that the CMS, identified as a key system underpinning self-evaluation, is dysfunctional in patients with schizophrenia, particularly dorso-medial PFC. This may have implications for lack of insight in schizophrenia. Hypofunction within the dorsomedial prefrontal region seems to be particularly important although other posterior and lateral cortical regions play a part and may modulate self-evaluative responses depending on the type of trait under consideration.

## Background

Impaired insight, defined as lack of acceptance of mental illness, inability to relabel pathological symptoms as abnormal and reluctance to accept treatment [1] is a fundamental feature of schizophrenia and related psychoses [2,3]. It is associated with more severe symptoms [4] and a range or poorer clinical and psychosocial outcomes [5,6]. While lack of insight is likely to have psychological and socio-cultural aspects, there are reasons to believe that it also possesses neurological underpinnings [2,7]. This is, in part, because several neuropsychiatric disorders are associated with marked impairments in self awareness and insight [8]. In addition, there is a small but reliable association between measures of poor insight and cognitive deficits – particularly executive functioning - in psychotic populations (see [9,10]).

However, the most recent contribution to this field comes from structural brain imaging in patients with schizophrenia or psychosis generally which have sought specific neurological correlates of poor insight. Most (although not all; [11]) MRI structural imaging studies of such patients have found significant relationships between lack of insight (variously defined) and a range of structural deficits (summarized in [12]). More recent sophisticated structural imaging studies have revealed relationships with specific brain regions or with fronto-temporal white matter [13]. The majority have found evidence for a relationship between poorer insight and either volumetric reduction or thinning of various cortical midline regions, most towards the anterior (frontal lobe: [14] medial-orbital prefrontal cortex PFC: [15]; medial PFC: [16,17]; medial-superior PFC: [18]; anterior cingulate: [15,19]; paracentral lobule: [16]) but also the posterior parts (posterior cingulate: [12,19]; precuneus: [18,20]; [16]). Two studies found poorer insight to correlate with *increased* volume in anterior midline regions [21,22]. Finally, a variety of other regions of reduced volume have also been implicated in the studies reviewed above including dorsolateral prefrontal cortex (DLPFC [23]), insula [24] and temporo-parietal regions [20].

The extent to which clinical insight is related to more general self-reflective and self-evaluative^1^ processes – part of metacognition [25] - is beginning to be addressed. This is important, not only because having an accurate representation of one’s traits, abilities and attitudes is essential to evaluating one’s own behaviour and hence adjusting it to social circumstances [26], but also because it provides a plausible normative framework within which to understand lack of insight in psychiatry. Such a framework would get round the problem of how to examine processes and models relevant to acceptance of mental disorder in a range of individuals.

The functional neuroanatomy of self-evaluation in healthy subjects has begun to be mapped [27]. In the most commonly used experimental paradigm, subjects are presented with a trait adjective and are asked whether it applies to them as opposed to another person (a friend, relative or famous personality). The results have been the subject of conceptual reviews and meta-analyses which demonstrate that a core set of regions – cortical midline structures (CMS) - are consistently engaged in tasks in which the self is the object of contemplation [28-31]; and this applies to mental states as well as personality characteristics [32]. The CMS comprises medial pre-frontal cortex (MPFC), posterior cingulate cortex (PCC) and anterior cingulate cortex (ACC). Self-evaluation can be broken down into several component processing steps such as directing attentional focus to oneself, followed by holding information in mind (working memory) in order to carry out a comparison with stored representations (episodic/autobiographical memory), all of which lead to a judgement or appraisal (executive functions). Hence, several brain regions commonly associated with component processes (eg DLPFC, medial temporal lobe; inferior parietal lobe, etc.) would be expected to play a role in such tasks [29,33]. Moreover there is clearly overlap between processes and networks which enable self and other evaluation, the precise extent of which is currently debated [26,31,33,34]. It has been claimed that that the greater the social distance between the self and the other, the more likely self activation regions will appear distinct ([35,36]; but see [37]).

Few functional imaging studies using fMRI have examined this issue in schizophrenia. Perhaps the first [38] showed a correlation between improvement in clinical insight scores with activation of medial PFC during an empathy task. However, Murphy et al. [39], were first to use a task that required participants to make decisions about the self-relevance of positive personality traits, and did not find significant differences in activation between schizophrenia patients and healthy controls. By contrast, Holt and colleagues [40] used a similar task (but with negative as well as positive personality traits) and found that patients displayed lower activation of the ventromedial PFC but higher activation of the median and posterior cingulate during self-evaluation compared to healthy controls; however, these authors did not examine the activity of brain regions outside of the cingulate. Modinos et al. [41] studied theoretically psychosis prone students with fMRI and found *increased* activation in CMS (plus insula) with a valenced self-reflection task compared to those less psychosis prone. Finally, work in a different diagnostic group, those with traumatic brain injury [42] showed increased activation in posterior and anterior CMS compared to controls, with activation correlated with insight into cognitive deficits.

The present study is the first to use whole-brain fMRI to examine the neural activity accompanying self-evaluation of illness traits as well as personality traits (both positive and negative) in schizophrenia patients and healthy controls and relating these to clinically rated insight. It was predicted that the schizophrenia patients would show reduced activation in anterior CMS during self-evaluation (given the structural abnormalities in this region) and that such activity would correlate with clinician-rated and self-rated assessments of insight. We also explored whether there would be other regions preferentially activated in such patients during self-evaluation suggesting compensatory or aberrant processing mechanisms particularly if correlated with insight scores.

## Methods

### Subjects

Eleven schizophrenia patients (Sz) were recruited from the Maudsley and Bethlem Hospitals and their affiliated clinics in London. Suitable patients were identified following consultation with their key workers and inspection of their medical records, and were eligible for inclusion if they had a primary diagnosis of schizophrenia (DSM-IV-TR criteria; American Psychiatric Association, 2000). Exclusion criteria were serious medical condition, brain damage, disability or substance abuse. Eight healthy controls (HC) were recruited via an advertisement placed in a London community centre, with respondents selected if they were aged between 18 and 65 years and reported having no current or previous psychiatric disorder. The same exclusion criteria applied. All patients had current or residual symptoms of hallucinations and delusions, and were receiving a fixed dosage of mainly atypical anti-psychotic medication at the time of testing. All participants gave written informed consent. The study was approved by the Institute of Psychiatry and Maudsley Hospital Research Ethical Committee [Ref 224/04 and 04/Q0706/114] and was in compliance with the Helsinki Declaration.

Demographic details on participants and clinical information on patients are displayed in Table 1. All participants were required to be native English speakers. Patients’ psychopathology was rated on the Positive and Negative Syndrome Scale (PANSS [43]). Insight was rated by NB (who was trained by the assessments’ author) following David’s scheme (see above) using the Schedule for the Assessment of Insight-expanded version (SAI-E; [44]), which has good reliability and validity [12]. Schizophrenia patients had a mean SAI-E total score of 16.2 (sd = 8.6; range = 4 to 28 out of a maximum of 28; higher SAI-E scores indicate better insight). Correlation with the PANSS ‘insight and judgement, item was r > .8 adding concurrent validity to the measure. We used insight total score rather than dimension sub-scores to limit the number of comparisons.

**Table 1:** Participant demographics and clinical details (± Standard Deviation)

	**Healthy controls. (HC; N = 8)**	**Schizophrenia patients (Sz; N = 11)**	**Group comparisons**
Mean Age (years)	31 (±9)	39 (±11)	NS
Gender (Male%)	3/5 (37%)	7/4 (64%)	NS
Mean Duration of Education (years)	16 (±3)	12 (±2)	t=3.63, *p* < .05
WASI *IQ* Score	112.4 (±16.4)	101.3 (±17.3)	F=4.21, *p* < .05
Digit span (forward + backward) (0–30)	18.1 (±4.74)	16.3 (±4.24)	NS
Trail making test:			
Ratio Score	2.45 (±1.51)	2.38 (±0.89)	NS
Mean Duration of Schizophrenia (years)	-	12 (±8)	
Patient Status(Inpatients/Outpatients)		4/7 (36%/64%)	
Insight (SAI-E) Total:		16.23 (±8.63)	
Illness Awareness		6.64 (±4.01)	
Symptom Relabelling		4.86 (±3.58)	
Compliance	-	4.73 (±1.79)	
PANSS score (SD)		82.0 (±16.4)	
- Insight (item G12)	-	3.18 (±1.78)	

### Neuropsychology

The following measures were administered; Parts A and B of the Trail Making Test [45]; forwards and backwards Digit Span subtests of Wechsler Adult Intelligence Scale (WAIS III; [46]). The Vocabulary and Matrix Reasoning subtests of the Wechsler Abbreviated Scale of Intelligence (WASI; [47]).

### FMRI task

Participants were shown trait adjectives and were asked to judge whether they applied to themselves, another person (Tony Blair, then British Prime Minister), or whether they contained the letter ‘a’. The trait adjectives were categorised as positive, negative, mental-illness related or physical-illness related. The valence of the trait types was unambiguous. Trait-types were matched on word length and where possible frequency of occurrence although data on frequency were not available on some words, particularly those that were illness related. Examples included: mental illness related: *Unstable, Crazy, Disordered, Psychotic…*; negative: *Evil, Cruel, Hostile, Dishonest, Selfish*…; positive: *Wonderful, Great, Special, Clever*…; physical illness related: *Diabetic, Cancerous, Paralysed…*etc. Mental and physical illness traits were rated similarly negative.

Each trait-type was divided into 3 matched groups of 8 traits, and the same group shown in three different contexts - self, other (Blair), or letter – giving 9 blocks per run. Context presentation-order was randomised as with run order, but remained the same for all runs done by the same participant, e.g. Self - Other - Letter order was unchanging for a given participant, but was reversed (etc.) for the next participant. Pilot work had shown that frequent switching of the object of the trait was confusing for participants. After one 3 block set was shown, the next 3 block set displays its constituent traits in a different order. No two consecutive blocks displayed the same traits. Thus 24 traits were shown 3 times per run = 72 traits seen per run.

Each block started with an orienting question: Are you…?/Is Tony Blair…?/Does the word contain the letter 'a'? This was shown for 6 seconds. Then each trait appeared following the probe question and remained on screen for 4.5 s regardless of when response was made followed by a 500 ms interstimulus interval. Participants responded by pressing one of two buttons: 'Yes/A bit' with the right middle finger or 'No, not at all' with right index finger. Again, pilot studies showed that this wording as opposed to a simple yes/no was optimal in countering a bias toward ‘no’ responses. Hence a block = 6 + 8x5 s = 46 s. So, each run = 9 blocks x 46 seconds per block = 414 seconds (6mins 54 seconds), with variable rest periods between runs. Participants were urged to respond to all items. If unsure about response, they were encouraged to reply as honestly as possible when ready, to what felt instinctively correct. Practise sessions were given before scanning using different trait terms. Response accuracy during letter-evaluation was used as a gauge of task adherence. Functional runs, each with a different trait-type (24 positive, 24 negative, 24 mental illness, 24 physical illness), were shown sequentially to each participant in one of 8 presentation-orders (orders that were evenly divided between the participants and matched between the two participant groups).

### FMRI acquisition

Gradient echo echoplanar imaging (EPI) data were acquired on a GE Signa 1.5 T system (General Electric, Milwaukee WI, USA) at the Maudsley Hospital, London. A quadrature birdcage headcoil was used for RF transmission and reception. 180 T_2_*-weighted images depicting BOLD contrast were acquired over each of 16 near-axial non-contiguous 7 mm thick planes parallel to the inter-commissural (AC-PC) line: TE 40 msec, TR 2 sec, in-plane resolution 3.44 mm, inter-slice gap 0.7 mm. In the same scanning session an inversion recovery EPI dataset was acquired at 43 near-axial 3 mm thick planes parallel to the AC-PC line: TE 73 msec, TI 80 msec, TR 16 s, in-plane resolution 1.72 mm, inter-slice gap 0.3 mm. This higher resolution dataset provided whole brain coverage and was later used to normalise the fMRI images acquired from each individual into standard stereotactic space.

### FMRI data analysis

#### Individual and group brain activation maps

Data were analyzed with the XBAM software developed at the King’s College London’s Institute of Psychiatry [48,49] (for a full description and references, see http://www.brainmap.it). The analysis is based on permutation testing that minimises assumptions. Our primary data were first processed to minimize motion related artefacts [50]. Subsequently, the data were smoothed using a Gaussian filter (FWHM 8.08 mm) to improve the signal to noise characteristics of the images. Experimental responses were analyzed by convolving each contrast of interest (self vs. other vs. letter (baseline)) with two gamma variate functions (peak responses at 4 and 8 sec). These two functions were chosen to encompass the known range of times to peak response following stimulus onset for BOLD effects. The best fit between the weighted sum of these convolutions and the time series at each voxel is computed using the constrained BOLD effect model [51]. Following computation of the model fit, a goodness of fit statistic is computed. This consists of the ratio of the sum of squares of deviations from the mean image intensity (over the whole time series) due to the model to the sum of squares of deviations due to the residuals (SSQ ratio). This statistic is used to overcome the problem inherent in the use of the F (variance ratio) statistic as the residual degrees of freedom are often unknown in fMRI time series due to the presence of colored noise in the signal.

Following computation of the observed SSQ ratio at each voxel, the data are permuted by the extensively characterized wavelet-based method [49]. Repeated application of this method at each voxel followed by recomputation of the SSQ ratio from the permuted data allows (by combination of results over all intracerebral voxels) the data-driven calculation of the null distribution of SSQ ratios under the assumption of no experimentally determined response.

The observed and permuted SSQ ratio maps for each individual were transformed into standard space [52] using a two stage warping procedure. For both stages of the warping process, a 12 parameter affine transform was employed. This involves first computing the average image intensity map for each individual over the course of the experiment. The transformations required to map this image to the structural scan for each individual and then from “structural space” to the Talairach template are computed by maximizing the correlation between the images at each stage. The SSQ ratio maps are transformed into Talairach space using these transformations. Group activation maps are computed by determining the median SSQ ratio at each voxel (over all individuals) in the observed and permuted data maps (medians are used to minimize outlier effects). The distribution of median SSQ ratios over all intracerebral voxels from the permuted data is then used to derive the null distribution of SSQ ratios and this can be thresholded to produce group activation maps at any desired voxel or cluster-level type I error rate. The detection of activated voxels is extended from voxel to 3D cluster level using well described methods [53]. In this two level clustering procedure the first (voxel-wise) thresholding is carried out at an uncorrected *p* value of .05 to give the maximum allowable sensitivity. In order to eliminate the resulting false positive activations, a second, cluster-level thresholding step is carried out and the threshold of this second step is adjusted to give an expectation of less than one false positive cluster over the whole brain. As the cluster level threshold is set at the whole brain level, the normal, voxel-wise issue of multiple comparisons does not apply. The computation of a standardized measure of effect SSQ ratio at the individual level, followed by analysis of the median SSQ ratio maps over all individuals treats intra- and inter-subject variations in effect separately. This constitutes a mixed-effect approach which allows for inferences from these results to be made about the larger population.

#### Analysis of behavioural data

A repeated measures analysis of variance (ANOVA) was performed to examine response choices and reaction times made to the different conditions, with a between-groups factor of participant-group (HC versus Sz), a within-groups factor of evaluation-level (self vs. other vs. letter), and a within groups factor of trait-type (positive versus negative versus mental illness versus physical illness). Reaction time data were trimmed to exclude responses above or below 2 standard deviations of that participant’s mean.

#### Analysis of imaging data

Voxel- and cluster-wise between-group differences in BOLD signal change were examined using a three-factor mixed analysis of variance (ANOVA) to examine response choices made to the different conditions, with a between-groups factor of participant-group (HC versus Sz), a within-groups factor of evaluation-level (self vs. other vs. letter). Trait types (positive, negative, mental illness and physical illness) were pooled initially in order to increase statistical power although post-hoc analyses were also undertaken to explore whether for example, there were specific effects of evaluating mental-illness related traits as opposed to generally negative traits or physical illness terms, and whether positive trait terms reveal different patterns of activation. Finally, significant and near significant interactions were re-analysed with trimmed reaction time used as a covariate. This did not materially alter the results (data not shown).

## Results

### Clinical data

Independent-samples t-tests revealed that schizophrenia patients had received significantly fewer years of education than healthy controls and had a lower current IQ although they were in the average range. The patients had a moderate degree of psychopathology, and intermediate levels of insight on the PANSS and SAI-E (Table 1).

### Behavioural data response type

There was no significant difference between the overall proportions of ‘Yes/A bit’ responses made by the two participant groups (Table 2). There was a significant difference between the responses made at each evaluation level (*F* (2, 26) = 62.2, *p* < .001) accounted for by significantly higher proportions made to *letter* trials vs. *self* and *other* trials, with no significant difference between the latter two. There was a significant effect of trait-type on the proportions of responses made (*F* (3, 39) = 37.9, *p* < .001) accounted for by significantly higher proportions of ‘Yes/A bit’ responses made to positive traits versus the other trait types. There was a significant 3-way interaction between trait-type, evaluation and group on the proportions of ‘Yes/A bit’ responses made (*F* (2.1, 26.7) = 3.58, *p <* 0.05) with schizophrenia patients making higher proportions to self-evaluated mental illness traits than the healthy controls, as would be expected (although such terms eg ‘crazy’ were accepted quite frequently by controls [54]).

**Table 2:** Upper Panel: Mean numbers of ‘Yes/A Bit’ Responses (0–1); Lower Panel: Mean response times (in msec) made by each participant group for each condition (Standard Deviation)

	**SELF-EVALUATED TRAITS**					**OTHER-EVALUATED TRAITS**					**LETTER-EVALUATED TRAITS**				
	**(Are you?*trait*)**					**(Is Blair?*trait*)**					**(Letter ‘a’?*trait*)**				
N° YES RESPONSES	Positive	Negative	Mental Illness	Physical Illness	All Traits	Positive	Negative	Mental Illness	Physical Illness	All Traits	Positive	Negative	Mental Illness	Physical Illness	All Traits
**HealthyControls** (*N* = 8)	0.72(0.45)	0.22(0.41)	0.05(0.22)	0.03(0.16)	0.28(0.45)	0.53(0.50)	0.35(0.48)	0.16(0.36)	0.00(0.00)	0.28(0.45)	0.53(0.50)	0.30(0.46)	0.49(0.50)	0.46(0.50)	0.44(0.50)
**Schizophrenia Patients** (*N* = 11)	0.36(0.48)	0.23(0.42)	0.25(0.43)	0.10(0.30)	0.24(0.42)	0.67(0.47)	0.17(0.38)	0.05(0.21)	0.10(0.30)	0.24(0.43)	0.52(0.50)	0.29(0.45)	0.47(0.50)	0.47(0.50)	0.44(0.50)
RESPONSE TIME															
**HealthyControls** (*N* = 8)	1417(717)	1262(526)	1089(503)	1038(475)	1218(589)	1414(620)	1478(627)	1200(525)	1116(464)	1321(589)	1069(373)	1036(348)	1042(402)	1094(513)	1057(402)
**Schizophrenia Patients** (*N* = 11)	1773(803)	1615(798)	1667(851)	1611(860)	1667(829)	1887(847)	1732(836)	1615(825)	1555(777)	1698(831)	1658(803)	1520(691)	1607(743)	1473(661)	1566(730)

### Reaction time

There was a significant difference between the overall response times made by the two participant groups (*F* (1, 13) = 7.12, *p <* 0.05), with the healthy controls making significantly faster responses (*M* = 1153 *ms*, *SE* = 158 *ms*) than the schizophrenia patients (*M* = 1669 *ms*, *SE* = 112 *ms*). There was a significant effect of evaluation level on response times (*F* (2, 26) = 5.38, *p <* 0.05) due to the *other* ≈ *self* > *letter* effect. There was no significant interaction with group. There was no significant difference between response times made to the different trait-types, no significant interaction with group or evaluation and no three-way interaction (see Table 2).

### FMRI data

#### Main effect of group

There were 5 clusters of activity relating to person evaluation (self- plus other-evaluation vs. baseline) where there was a significant effect of group (i.e. diagnosis).

#### Healthy controls > schizophrenia patients

**Figure 1:**
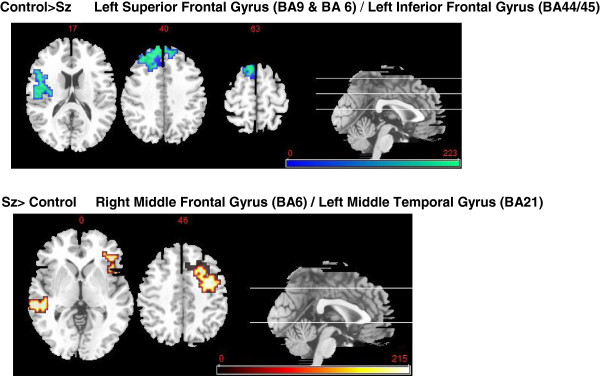
**Blood oxygen level dependent activation maps.** Upper Panel (cold colours): clusters of activation during person-evaluation where healthy controls (HC) were significantly greater than schizophrenia (Sz) patients. Lower Panel (warm colours): clusters of activation during person-evaluation where Sz were significantly greater than HC (see Table 4). Right side of axial image is right side of brain.

Controls had significantly (*p* < .0006) and near-significantly (*p* < .0081) higher activation of three areas during person-evaluation (self- and other-evaluation combined vs. ‘baseline’ letter task) of the combined trait-types than the patients (significance levels adjusted for whole brain cluster-level comparisons. Figure 1/Table 3 upper panel): These areas were centred on the left superior and inferior frontal gyri spanning dorso-medial and ventro-lateral prefrontal cortex.

**Table 3:** **Group comparison for self and other evaluation: all traits combined (see Figure**1**)**

**Cerebral region**	**Peak talairach coordinates x, y, z*(range)***	**3-D Cluster size (in voxels)**	**Significance level**
Healthy Controls (HC) > Schizophrenia patients (Sz)			
**Left Superior Frontal Gyrus, BA9**	-6, 53, 32(x: -6 - -14, y: 30–53, z: 22-42; includes BA 8)	153	*p* = 0.0006
**Left Inferior Frontal Gyrus, BA44/45**	-48, 16, 16(x: -41 - -48, y: -4–16, z: 9–16; includes insula)	73	*p* = 0.0027
**Left Superior Frontal Gyrus, BA6**	-6, 25, 54	20	*p* = 0.0081^#^
Schizophrenia patients (Sz) > Healthy Controls (HC)			
**Right Middle Frontal Gyrus, BA6 Right Middle Frontal Gyrus, BA45**	35, -1, 40(x: 29–37, y: -1 –19, z: 40-54; includes BA 8, 9)	121	*p* = 0.0005
	51, 27, 8(x: 38–49, y: 5–41, z: -7 –29; includes BA 46,47)	148	
**Left Middle Temporal Gyrus, BA21**	-58, -26, -7	26	*p* = 0.0083^#^

^#^Failed to reach significance after adjustment for cluster level analysis.

BA: Brodmann Area.

#### Schizophrenia patients > healthy controls

Schizophrenia patients had significantly (*p* < .0005) higher activation in a large cluster with two constituent areas during person-evaluation (self-evaluation and other-evaluation combined) of the combined trait-types than the HC (Figure 1/Table 3 lower panel). This centred on the right middle frontal gyrus (DLPFC), encompassing BA 6 and BA 45. There was also a near significant cluster in left middle temporal gyrus (*p* = .008).

**Table 4:** **Underactive regions during self-evaluation (vs. other-evaluation) in patients (vs. controls): group x level interaction (Figures**2**and**3)

**Cerebral region**	**Peak talairach coordinates x, y, z*(range)***	**3-D Cluster size (in voxels)**	**Significance level**
**COMBINED TRAITS**			
Left Superior Frontal Gyrus, BA9	-6, 53, 32(x: -6 – 19, y: 37-53, z: 27-42 includes BA8)	31	*p* = 0.0006
Right Superior (& Medial) Frontal Gyrus, BA9			
		26	
Right Posterior Cingulate, BA23*	-1, -55, 16(x: -3 – 7, y: -51-56, z: 16-26 includes L post. cingulate)	26	*p* = 0.0053
POSITIVE TRAITS			
Right Medial Frontal Gyrus, BA9	4, 48, 26	39	*p* = 0.0012
NEGATIVE TRAITS			
Left Superior Frontal Gyrus, BA9	-4, 48, 31	42	*p* = 0.0016
MENTAL ILLNESS TRAITS			
Right Inferior Parietal Lobule, BA40	47, -33, 37	13	*p* = 0.0059^#^
PHYSICAL ILLNESS TRAITS			
Right Middle Occipital Gyrus, BA18/19	33, 82, 8	56	*p* = 0.0007

*Became significant after analysis with response time as a co-variate.

^#^Cluster level significance level, p<.005. BA: Brodmann Area.

#### Group x Level Interaction (self vs other) Healthy controls > schizophrenia patients

**Figure 2:**
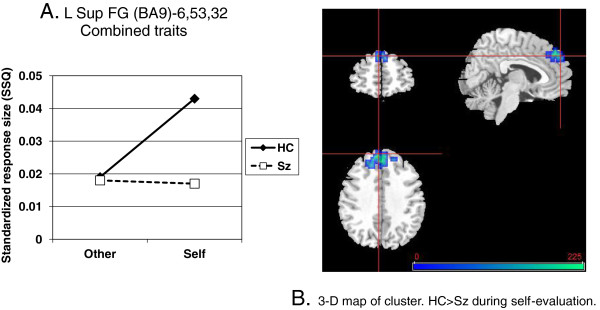
**A. Regional cluster in left superior frontal gyrus (57 voxels) showing relative underactivity in schizophrenia patients (Sz) versus healthy controls (HC) during self-evaluation (vs other (Blair)) of traits. B**. Cluster map (see Table 4). Right side of axial/coronal image is right side of brain.

Of most interest was the interaction between diagnosis and self (versus other) processing with all traits combined. This produced just one highly significant cluster in the dorsomedial prefrontal cortex (left superior frontal gyrus, BA9; *p* = .0006 adjusted; see Figure 2. Table 4).

**Table 5:** **Overactive regions during self-evaluation (vs. other-evaluation) in patients (vs. controls): group x level interaction (Figures**4**and**5**)**

**Cerebral region**	**Peak talairach coordinates x, y, z*(range)***	**3-D Cluster size (in voxels)**	**Significance level**
**POSITIVE TRAITS**			
Right Anterior Cingulate, BA32	8, 45, -6	57	*p* = 0.0017
Right Precuneus, BA7	18, -70, 42	17	*p* = 0.0056
Left Precuneus, BA7	-14, -59, 48	21	*p* = 0.007^#^
**NEGATIVE TRAITS**			
**No interaction found**			
**MENTAL ILLNESS TRAITS**			
Left Superior Frontal Gyrus, BA8*	-6, 31, 47(5, 21, 47 inc. R med frontal gyrus)	30	*p* = 0.0033
Right Middle Frontal Gyrus, BA10	38, 50, 0	19	*p* = 0.007^#^
**PHYSICAL ILLNESS TRAITS**			
Left Anterior Cingulate, BA32	-15, 42, -6	70	*p* = 0.0006
Right Inferior Parietal Lobule, BA40	50, -30, 25	28	*p* = 0.0021
Left Inferior Parietal Lobule, BA40 / Left Postcentral Gyrus, BA2	-41, -26, 30	31	*p* = 0.0024

^*^Became significant after analysis with response time as a co-variate.

^#^Cluster level significance level, p<.005. BA: Brodmann Area.

**Figure 3:**
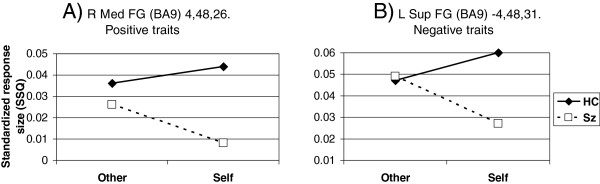
**(left). Regional cluster showing relative over-activity in schizophrenia patients (Sz) versus healthy controls (HC) during self-evaluation of mental illness traits (self vs. other (Blair)). B** (right column). Pearson’s correlation with insight scores (Sz only) and scatter plot (see Table 4).

When individual traits were considered, other adjacent dorsomedial prefrontal regions emerged for positive and negative traits (Table 4). An interaction indicative of relatively lower activity during mental illness trait self-evaluation in schizophrenia was of borderline significance (*p* < 0.0059) in one area: right inferior parietal lobule (BA40); peak Talairach coordinates, 47, -33, 37 (see Figure 3). Finally, there was a cluster in right middle occipital gyrus which yielded a significant self by diagnosis interaction for physical traits.

#### Schizophrenia patients > healthy controls

**Figure 4:**
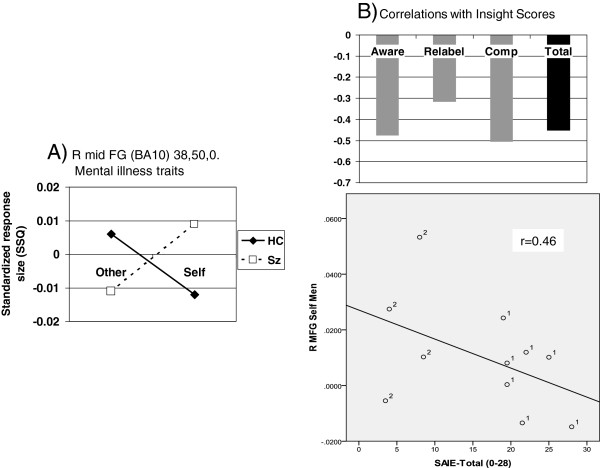
**A (left). Regional cluster showing relative over-activity in schizophrenia patients (Sz) vs. healthy controls (HC) during self-evaluation of positive traits (self vs. other (Blair)). B** (right column). Pearson’s correlation with insight scores (Sz only) and trait ownership (Sz and HC) and scatter plot (all participants) (see Table 5).

There were no regions detected of significantly increased activation in the schizophrenia patients versus healthy controls in the combined trait analysis for self versus other. Exploratory analyses when individual traits were considered showed however, two regions of interest: the left superior frontal gyrus (BA 8, peak Talairach coordinates: -6,31,47; *p* < .003), superior and more caudal than the region which showed significantly reduced activation for self versus other, and the right middle frontal region (ventro lateral PFC; *p* < .007) which was near significant after adjustment (Figure 4A). Self evaluation of positive traits activated anterior central midline structures (right ACC) more so in patients, and more posterior regions (precuneus, BA7). Finally, self evaluation of physical illness traits produced significantly higher activation in the anterior cingulate and left and right inferior parietal lobule in patients (Table 5).

**Figure 5:**
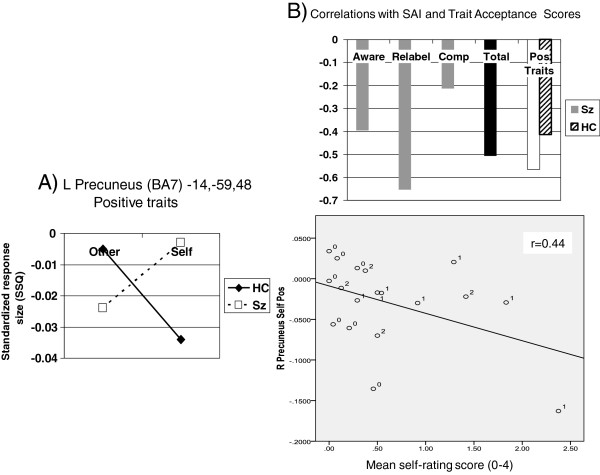
**Regional clusters showing relative underactivity in schizophrenia patients (Sz) versus healthy controls (HC) during self-evaluation of traits.A**. Positive traits (self vs. other (Blair)). **B**. Negative traits (self vs. other (Blair) see Table 5).

#### Correlations between insight, trait-acceptance and self-relevant activation

Correlational analyses were performed on regions identified as potentially relevant to self-evaluation in patients versus controls (all traits combined) to explore associations with insight scores. Reduced activity in the left superior frontal gyrus which showed a significant group by level (self vs. other) interaction, showed a correlation coefficient with the awareness of illness subscale of the SAI-E insight scale of r = 0.43 (p < .05), and lower acceptance of negative traits, but only 0.12 with the total score.

The schizophrenia patients showed relative overactivity during mental illness trait self-evaluation in the right middle frontal gyrus (BA10; peak Talairach coordinates, 38, 50, 0). Higher activity here was moderately correlated with *lower* SAI-E insight ratings (Figure 4B).

Higher precuneus activity (both left and right) was moderately-strongly correlated with lower insight ratings. Interestingly, trait ownership scores were also found to correlate negatively with activity in this region in both patients *and* controls, i.e. the greater the activity in the left precuneus, the less positive traits were endorsed as self relevant (Table 5; Figure 5A and B).

## Discussion

To our knowledge, this is the first study to have examined whole brain activity with fMRI during examination of trait adjectives including those related to being ill, in relation to self or other in schizophrenia patients. The self-evaluation paradigm has become well established in the social cognition literature and appeared to be applicable to patients with psychiatric disorder. We found clusters of reduced activation in the patients - in left dorsomedial PFC - in relation to person evaluation including those within the CMS identified in previous studies and meta-analyses [26,29,31]. The left inferior frontal area was also hypoactive, possibly reflecting the linguistic burden of the task [55]. More lateral and dorsal frontal regions showed relative increases in activation (also self processing areas, see [28]) in the patient group and a left middle temporal region which failed to reach significance at the cluster level, perhaps representing compensation strategies. In the combined analysis, no posterior regions emerged such as the posterior cingulate and precuneus (or indeed regions such as amygdala and insula). This may have been due to similar activation in these areas in response to both person evaluation and baseline trials. However, our focus was on areas where there was a difference between patients and controls which might then be related to self processing, psychosis and insight.

We predicted that the schizophrenia group would show reduced activation during self-evaluation in regions within the CMS. This was supported with a single cluster of voxels within the main medial frontal region which showed a significant interaction between diagnostic group and self-evaluation when responses during all the trait types were combined, thus giving the most robust data (see Figure 2). A second region of significant interaction was also noted in the right posterior cingulate, a region well established in the self-appraisal literature [29,30,56] after adjustment for reaction time. The PCC appears to be important in experiential self reflection [56] and was also highlighted as a region of reduced grey matter density in relation to the symptom relabeling dimension of insight [12]. The medial frontal locus is somewhat more superior (z = 32) than the main focus derived from meta-analyses (z = 6; [31]) although consistent with the self-appraisal imaging literature [29,30,54]. The middle-posterior cingulate was shown by Holt et al. [40] to be more activated in schizophrenia patients than controls in a similar self-reflection paradigm. They went on to show abnormal connectivity between this region and more anterior parts of the cingulate, which was also ‘overactive’ in our patients during self evaluation of specific traits. Exploratory analyses were carried out to look at cerebral responses to self-evaluation of individual traits and correlation with insight, which was also part of the hypothesis under test. Interactions indicated that the patients were particularly hypoactive during self-evaluation in the right medial frontal gyrus (positive traits), left superior frontal and superior temporal gyrii (negative traits) and right inferior parietal lobule (BA40; mental illness traits). There was no obvious pattern to indicate that the valence of traits was decisive in determining the locus of activation. Lower activity in all these areas except the right medial frontal gyrus tended to show weak-to-moderate correlations with lower SAI-E insight in the patients (data not shown). Interactions indicated that the patients were particularly hyperactive during self-evaluation in the right anterior cingulate and bilateral precuneus (positive traits), right middle frontal gyrus (BA10; mental illness traits), and left inferior parietal lobule (BA40; physical illness traits). *Higher* activity in all these areas showed a correlation with *lower* SAI-E insight in the patients and this was significant in the right middle frontal gyrus for mental illness traits and the left precuneus for positive traits (Figures 4 and 5). Such activity was also related to higher endorsement of positive traits in both healthy controls and patients which is consistent with the observation that grandiosity and lack of insight frequently go together [44,57,58].

Activation in the non-dominant parietal lobe is of interest since damage to this region is a frequent finding in neurological patients with anosognosia [59] and has been linked with disorders of awareness in schizophrenia [60]. While hypoactivation (in relation to mental illness traits) was noted, so too was bilateral inferior parietal (plus somatosensory cortex and ACC) hyper-activation in relation to physical illness terms which could plausibly be interpreted as correlating with ‘somatic insight’. While relative over- and under-activation should be interpreted cautiously it may be tempting to consider lack of insight as having both deficit (failure to engage in self-evaluation) and excess (active ‘denial’) elements. Correlating fMRI activation and the clinician rated insight score is a partial validation of the method used to elicit self-appraisal of mental disorder although we were not in a position to validate or confirm the participants’ accuracy of self-appraisal of personality traits.

However, we suggest that the main finding of reduced dorso-medial PFC activation in patients versus controls is best explained by a failure to carry out effective self-evaluation and not merely a reluctance to engage in the activity. This correlated with the symptom awareness component of the insight scale particularly although other components weakly related. Our own work (with these patients [54,61]) and that of others using subjective reports of self-reflection does not paint a picture of lack of effort to understand the self in schizophrenia but rather a failure to reach satisfactory conclusions or at least conclusions which accord with the consensus judgements of others [61-63]. In acquired brain injury it appears that CMS activity is *increased* during attempts to self-reflect (compared to controls) and that the greater activation, the greater the insight [42]. An analogous pattern is seen in healthy students who are theoretically psychosis prone [40], as if to say these states place greater demands on self-evaluation systems. We propose that with established schizophrenia the ability to self-evaluate effectively, diminishes [64] and this is mirrored in reduced brain activation in key structures underpinning the function. The possibility that this may recover with recovery of the illness forms an intriguing hypothesis (see [38]) and would be consistent with insight having both state and trait components [65]. The precise nature and content of ‘generic’ self-evaluation is presumably modulated by other brain systems, as evidenced by patterns of increased and decreased activation on fMRI in posterior cerebral regions (PCC, precuneus, occipital cortex) and other more lateral frontal, parietal and temporal regions. Inferior parietal, precuneus and right middle frontal regions providing mental-illness-specific ‘fine tuning’.

The functional abnormalities discussed above were specific to self-evaluation. However the medial PFC is consistently implicated in both structural and functional abnormalities in schizophrenia per se with multimodal imaging techniques including diffusion tensor imaging, voxel based morphometry and fMRI [66]. This background plus the specific structural MRI findings in relation to insight [12,14,18] speak to the ‘trait’ aspects of poor insight and its associated cognitive deficits [9,67,68]. The patients in the current study had slightly lower IQ than controls although they showed well preserved functioning on working memory and set shifting, so cognitive impairment is unlikely to explain the results to a large extent. Nevertheless, the partial structural brain basis for poor insight in schizophrenia may explain why correlations with insight scores were not strong in relation to the locus of diminished medial PFC activation in the current fMRI study, since variation in BOLD signal is presumably limited by the neural substrate.

### Limitations

There are a number of limitations to this study. Most important is the small sample size precluding a more thorough exploration of the influence of clinical variables on the findings. Replication with a larger sample is warranted. Related to this is the imperfect matching of cases and controls. Lower current IQ and slower reaction time are almost invariable in the schizophrenia research literature hence ‘controlling’ for them, even if possible, may not be valid. Furthermore, patients were all on regular medication which may have confounded the results directly or by reducing motivation and volition, but again this is not unusual in the field. It may be somewhat reassuring that we recently found that atypical antipsychotic mediation tended to ‘normalise’ medial PFC activation in an emotion recognition task [69]. Finally, the optimal choice of ‘other’ in self-other comparisons is not clear from the literature [32,37]. In line with previous seminal work [27], Prime Minister Blair was chosen since he was very familiar to all participants, although the strong feelings that he evoked in some may have complicated the results. Choosing say, the participant’s mother or best friend might also have lead to unpredictable emotional reactions. Finally the precise linkages between self-evaluation, self-appraisal and insight need to be further addressed in cognitive and neuroimaging studies.

## Conclusions

We have demonstrated using fMRI that the CMS identified as a key system underpinning self-evaluation, is dysfunctional in patients with schizophrenia, particularly dorso-medial PFC. Its precise relevance to insight is likely to be complex. First of all, insight is clearly a biopsychosocial construct [2,70]. Second, the neurocognitive sub-components of insight have not been fully mapped out although some preliminary models have been put forward [31]. The current study suggests that lack of insight involves both abnormal decreases in activation in medial frontal brain networks, as well as increases in activation, within CMS.

## Endnote

^a^‘Self-evaluation’ is the term that perhaps comes closest to describing the psychological process of interest, although in practise it is used interchangeably with the terms ‘self-appraisal’, ‘self-assessment’ and ‘self-reflection’.

## Competing interest

The authors have no conflicts of interest to declare.

## Authors’ contributions

NB developed the test materials, tested participants, collected and analysed the data. VG and MJB supervised the imaging work and data analysis. SS assisted with data analysis and interpretation. ASD conceived the study and managed all aspects of the project and drafted and approved the final manuscript. All authors had input into the manuscript.

## Pre-publication history

The pre-publication history for this paper can be accessed here:

http://www.biomedcentral.com/1471-244X/12/106/prepub
